# Depressive Symptoms in Patients with Fibromyalgia: Current Evidence and Preventive Approaches

**DOI:** 10.3390/jcm12123987

**Published:** 2023-06-12

**Authors:** Shuo-Yan Gau, Tsung-Hsuan Hung, Min-Fei Chuang, James Cheng-Chung Wei

**Affiliations:** 1 School of Medicine, Chung Shan Medical University, Taichung 402, Taiwan; 2 Department of Medical Education and Research, Kaohsiung Veterans General Hospital, Kaohsiung 813, Taiwan; 3 School of Medicine, National Cheng Kung University, Tainan 701, Taiwan; 4 Kaohsiung Chang Gung Memorial Hospital, Kaohsiung 833, Taiwan; 5 Department of Neurology, Chung Shan Medical University Hospital, Taichung 402, Taiwan; 6 Department of Allergy, Immunology & Rheumatology, Chung Shan Medical University Hospital, Taichung 402, Taiwan; 7 Graduate Institute of Integrated Medicine, China Medical University, Taichung 402, Taiwan; 8 Institute of Medicine, Chung Shan Medical University, Taichung 402, Taiwan

Fibromyalgia is a chronic inflammatory disease characterized by multifocal pain, fatigue, and cognitive impairment [1]. Despite the prevalence of fibromyalgia ranging from 2% to 4% in epidemiological studies, accurate diagnosis remains challenging, potentially leading to the underestimation of its actual prevalence [2]. Fibromyalgia is often accompanied by comorbidities in various systems, including autoimmune diseases [3,4], psychiatric disorders [5,6], and central nervous system dysfunctions [7,8]. The issue of psychiatric comorbidity status in fibromyalgia has long been discussed since the onset of a psychiatric disorder could massively influence the patient quality of life, in turn worsening the prognosis of fibromyalgia [9].

Depression is a common comorbidity of fibromyalgia, and various studies indicate high prevalence rates. A meta-analysis demonstrated that approximately one-fourth of fibromyalgia patients have comorbid depression, while more than half experience a major depressive disorder at some point in their lives [10]. The relationship between fibromyalgia and depression is bidirectional, with a genetic predisposition to depression slightly increasing the risk of fibromyalgia and fibromyalgia greatly increasing the risk of depression [11]. While the exact pathophysiological mechanisms linking depression and fibromyalgia are not yet fully understood, two critical genes, SLC6A4 and COMT, have been identified as potential factors that massively affect the interplay between the two diseases. SLC6A4 is responsible for encoding the serotonin transporter, which may increase the susceptibility to fibromyalgia by influencing serotonin reuptake [12]. The COMT gene has been implicated in dopamine breakdown in the prefrontal cortex, which is responsible for psychological disorders such as depression and anxiety [13]. Proinflammatory cytokines, including IL-6 and IL-8, have been found to be higher in patients with fibromyalgia compared to healthy controls. The level of these cytokines correlates with the severity of fibromyalgia symptoms [14,15]. In addition, IL-6 is associated with fatigue, painful events, stress, and depression, while a high level of IL-8 can induce neuroinflammation, which is involved in multiple psychiatric disorders [15,16]. The evidence above suggests a complex relationship between fibromyalgia, depression, and cytokines, but the exact mechanisms have not been firmly established.

Central sensitization, characterized by increased nociceptive pathway function and reduced pain threshold, has been proposed as a mechanism underlying the association between fibromyalgia and depression [17,18]. Abnormalities in the somatosensory nervous system contribute to the inhibition of neural injury and inflammation, potentially leading to the manifestation of depressive symptoms in patients with fibromyalgia [17]. Clinical studies have provided evidence of abnormal changes in nociceptive processes in fibromyalgia patients, supporting the central sensitization hypothesis [19,20]. In clinical studies, central sensitization was reported to be associated with depressive symptoms in diseases presenting chronic pain [21]. Moreover, in addition to depression, central sensitization was also believed to be involved in the onset of various psychiatric situations, such as anxiety, anger, and hypervigilance, due to overlapping pathogenesis mechanisms [18,22]. However, the current evidence lacks large-scale, lab-based, and real-world studies with high evidential power, necessitating further research to fully elucidate the role of central sensitization in the development of depressive symptoms in fibromyalgia patients.

In recent years, researchers have explored different approaches to address the psychological issues in patients with fibromyalgia and improve their quality of life, such as medical interventions and changes in lifestyle. However, though several meta-analyses to date have provided some integrated evidence regarding the efficacy and safety of interventions, currently there have been no trials comparing the influence of these interventions in head-to-head study designs.

## 1. Medications: Antidepressants

When evaluating the treatment indicators of pharmacological approaches for fibromyalgia, the evaluation of psychiatric statuses is critical [23]. In a recent network meta-analysis by Farag et al., 2022, the efficacy and acceptability of different medications for fibromyalgia were compared, including pregabalin, duloxetine, milnacipran, and amitriptyline, the only medication approved for fibromyalgia by the U.S. Food and Drug Administration. The study incorporated the results of 36 randomized controlled trials involving 11,930 fibromyalgia patients and evaluated psychiatric indicators such as depressive symptoms and fatigue. The findings indicated that among the medications compared, duloxetine 120 mg showed the greatest improvement in pain relief and alleviation of depressive symptoms. However, when compared to placebos, none of the four drugs of interest showed significant efficacy in preventing subsequent depression in fibromyalgia patients [24].

## 2. Lifestyle Change: Exercise

Exercise is commonly recommended as an effective non-pharmacological treatment, with a few systematic reviews reviewing the efficacy of different types of exercise, which highlighted its efficacy in alleviating pain and improving physical function to some extent [25,26,27,28]. Couto and Monteiro conducted a comprehensive meta-analysis that examined the effectiveness of three types of exercise: aerobic exercise, resistance exercise, and stretching exercises [29]. The study included 18 RCTs with 1184 fibromyalgia patients. The integrated results showed that three types of exercises have a significant and large effect on pain reduction. Regarding the onset of depression, only aerobic exercise demonstrated a moderate and significant impact in the experimental group, while no significant differences were found among the three types of exercise. It is important to note that the quantity and intensity of exercise should be tailored to the individual capacity and the guidance of a physical technician is crucial to prevent the exacerbation of symptoms and resistance to exercise.

## 3. Nutrient Supplement: Vitamin D

Lack of vitamin D has been suggested as a risk factor for the development of chronic pain. A European longitudinal study reported that for men with fewer than 15.6 ng/mL of vitamin D in serum, the risk of widespread chronic pain was increased significantly when compared with those with higher serum vitamin D concentration, with an odds ratio of 1.93 [30]. Similar findings were also observed in a study based on the female population [31]. Recently, studies have proposed that vitamin D supplementation could potentially reduce the risk of psychiatric symptoms in fibromyalgia patients and enhance patients’ quality of life [32,33]. However, though positive results were reported in single studies, the integrated evidence indicated that a significant improvement in Beck’s Depression Inventory (BDI) scores was not observed in fibromyalgia patients with sufficient vitamin D supplementation [34]. Regarding the effect of vitamin D and the depressive symptoms in fibromyalgia, the current evidence was massively limited by small sample sizes and high heterogeneity. Therefore, though supplementation with vitamin D has been reported to have a positive influence on some specific indicators of fibromyalgia (i.e., Fibromyalgia Impact Questionnaire (FIQ) scores or social functions) [34], the efficiency should be prudently interpreted based on current evidence.

## 4. Psychological Interventions

Psychological interventions are considered potential novel approaches for the depressive symptoms of fibromyalgia patients. For example, Cognitive Behavior Therapy (CBT) is one of the mainstream psychotherapies used to treat people with chronic pain. The primary orientation of CBT is to make people change negative thoughts and modify behaviors to deal with pain problems [35]. It has been found effective in reducing pain, alleviating depressive mood, and improving sleep problems in individuals with chronic pain symptoms [36]. In the case of fibromyalgia, CBT interventions have shown a significant reduction in the onset of depressive symptoms [37]. Additionally, a recent randomized controlled trial conducted by Serrat et al. (2021) compared a multicomponent treatment, including pain neuroscience education, therapeutic exercise, cognitive behavioral therapy, and mindfulness, with usual therapies. The results suggested that the multicomponent treatment may be more effective in addressing depressive symptoms in fibromyalgia patients [38]. However, it is important to consider that accessibility and insurance coverage for CBT may vary in different countries.

Depressive symptoms significantly impact the quality of life and prognosis of fibromyalgia patients. Therefore, the development of novel management approaches is crucial. Researchers and clinicians in the fields of immunology and psychiatry should conduct future studies with larger sample sizes to provide more robust evidence on the efficacy of current approaches in addressing depressive symptoms in people with fibromyalgia.

## Data Availability

Not applicable.
